# Supplementary material for: Cellular Automata Inspired Multistable Origami Metamaterials for Mechanical Learning
Source: Adv Sci (Weinh). 2023 Oct 23;10(34):2305146. doi: 10.1002/advs.202305146 (PMC10700163; doi:10.1002/advs.202305146)
Supplement: Supplementary file 1 — Supporting Information [file ADVS-10-2305146-s003.pdf]

ADVANCED SCIENCE
Open Access

Supporting Information

for *Adv. Sci.*, DOI 10.1002/advs.202305146

Cellular Automata Inspired Multistable Origami Metamaterials for Mechanical Learning

Zuolin Liu, Hongbin Fang, Jian Xu and Kon-Well Wang*

Supporting Information

Cellular automata inspired multistable origami metamaterials for mechanical learning

Zuolin Liu, Hongbin Fang, Jian Xu, and K.W. Wang*

Zuolin Liu, Hongbin Fang, Jian Xu
Institute of AI and Robotics, State Key Laboratory of Medical Neurobiology, MOE Engineering Research Center of AI & Robotics, Fudan University, Shanghai 200433, China
E-mail: fanghongbin@fudan.edu.cn

Zuolin Liu, K.W. Wang
Department of Mechanical Engineering, University of Michigan, Ann Arbor, MI 48109, USA

S1. Modeling of the SMO metamaterial

The kinematics of a single SMO cell can be characterized by two different types of parameters. One of them is the geometry parameters of the constituent Miura-ori sheets, i.e., the crease lengths (a_s, b_s) and the sector angles (γ_s), where the subscript s denotes the two different Miura-ori sheets ($s=\alpha,\beta$). To ensure stacking, b_α should be equal to b_β, i.e., $b_\alpha=b_\beta=b$. Besides, kinematic compatibility requires $a_\alpha\cos\gamma_\alpha = a_\beta\cos\gamma_\beta$. The other type of parameter is the folding angles θ_s, which are the dihedral angles between the sheets and the $x-y$ plane. It can be utilized to characterize the folding of a unit cell. For the rigid folding scenario, i.e., the facets are rigid and the creases act like hinges, the SMO unit cell is a single-degree-of-freedom system. Therefore, the folding angle θ_α can be uniquely used to characterize the folding motion of a unit cell. Other dihedral angles ρ_i ($i=0,1,\cdots,4$) (shown in Figure S1(A)) can be expressed as functions of this independent variable θ_α:

$$\begin{aligned}&\theta_\beta=\arccos\left(\cos\theta_\alpha\tan\gamma_\alpha/\tan\gamma_\beta\right),\rho_0=\theta_\beta-\theta_\alpha,\rho_1=2\arccos\left(\frac{\sin\theta_\beta\cos\gamma_\beta}{\sqrt{1-\sin^2\theta_\beta\sin^2\gamma_\beta}}\right),\\&\rho_2=\pi-2\theta_\beta,\rho_3=2\arccos\left(\frac{\sin\theta_\alpha\cos\gamma_\alpha}{\sqrt{1-\sin^2\theta_\alpha\sin^2\gamma_\alpha}}\right),\ \rho_4=\pi-2\theta_\alpha.\end{aligned} \tag{1}$$

Two topological different configurations are identified with $\theta_\alpha<0$ and $\theta_\beta>0$ by reassigning the mountain and valley creases of Miura-ori sheet α, which are interpreted as ‘0’

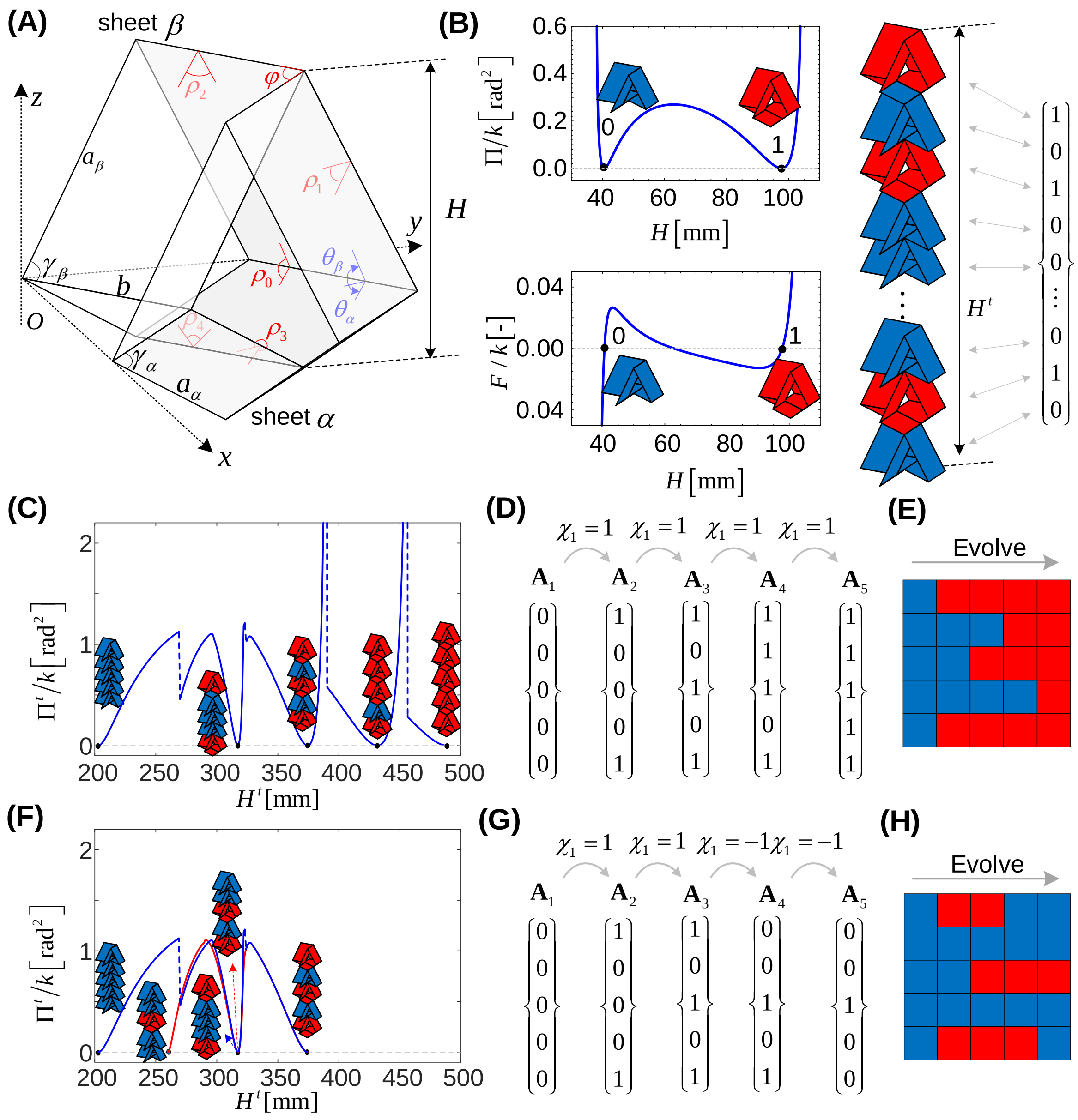

Figure S1. Potential energy landscape with respect to the external control and the corresponding mechanical signal streams. (a) and (b) show the potential energy landscape and the mechanical signal streams with an actuation rule $\boldsymbol{\chi} = [1 \quad 1 \quad 1 \quad 1]^T$; while (c) and (d) present them with an actuation rule $\boldsymbol{\chi} = [1 \quad 1 \quad -1 \quad -1]^T$.

and '1' states for an origami mechanical bit, respectively, see Figure S1(B). To distinguish, we present the configurations with different colors.

By assigning torsional stiffness per unit length k for the crease in sheet β, the elastic potential energy of the unit cell can be written as

$$\Pi = ka_\beta \left(\rho_1 - \rho_1'\right)^2 + kb\left(\rho_2 - \rho_2'\right)^2, \tag{2}$$

where ρ_1' and ρ_2' corresponds to the dihedral angle of the stress-free stable configuration, which can be obtained by substituting the stress-free folding angle (denoted as θ') to Equation

(1). Since ρ_1 and ρ_2 are even functions of the folding angle θ_α, the structure can have another stable configuration with $\theta_\alpha = -\theta'$.

The external height of a unit cell can also be expressed as a function of the folding angle θ_α:

$$H = a_\alpha \sin\theta_\alpha \sin\gamma_\alpha - a_\beta \sin\theta_\beta \sin\gamma_\beta. \tag{3}$$

In what follows, the geometry parameters are adopted as

$$b = 38.1\text{mm}, a_\alpha = 38.1\text{mm}, a_\beta = 1.25a_\alpha, \gamma_\alpha = 60^\circ, \theta' = -\pi/3. \tag{4}$$

The potential energy landscape with respect to the external height present double wells (top panel in Figure S1 (B)), which reals the bistable nature of a single unit cell. The two stable configurations correspond to the two binary states of a mechanical bit, with which information can be stored. By taking the derivative of the potential energy with respect to the external height, the force-displacement relation can be obtained as $F = \mathrm{d}\Pi/\mathrm{d}H = \left(\mathrm{d}\Pi/\mathrm{d}\theta_\alpha\right)/\left(\mathrm{d}H/\mathrm{d}\theta_\alpha\right)$. We illustrate the force-displacement curve in the bottom panel in Figure S1(C), the two stable configurations are the intersection points with the axis $F = 0$ and with positive slopes. Note that the two states are with different external heights, i.e., the height of state '1' is larger than that of state '0', therefore we can realize the snap-through transition of these two stable configurations by changing the external height, i.e., rewrite the storage of the mechanical bit with height control.

For an origami chain structure with J Miura-ori unit cells connected in series, see the right panel in Figure S1(B), the independent variable θ_α in j^{th} unit cell is denoted as θ^j. The vector consisting of all independent variables is denoted as $\boldsymbol{\theta}$. Similarly, the other dihedral angles, elastic potential energy, external height, and the torsional stiffness per unit length are denoted as ρ_i^j, Π^j, H^j and k^j ($i = 0,1,\cdots,4$, $j = 1,2,\cdots,J$), respectively. Besides the elastic potential energy within each unit cell, extra potential energy is induced by the zig-zag connection between the adjacent cells. Define the dihedral angle corresponding to the connecting crease in j^{th} unit cell as $\varphi^j = 2\tan^{-1}\left(\cos\theta^j \tan\gamma_1\right)$, ($j = 2,3,\cdots,J$), the extra potential energy can be written as

$$\Pi_c^j = \frac{1}{2}k_c^j b\left(\varphi^j - \varphi^{j-1}\right)^2, \tag{5}$$

where k_c^j is the equivalent stiffness per unit length corresponding to the strength of the deformation coupling from the zig-zag connecting creases between cell j and cell $j-1$. Therefore, the total potential energy of the origami chain structure is the summation of the elastic folding energy of every individual unit cell and the potential from their coupling, i.e.,

$$\Pi^t = \sum_{j=1}^{J}\Pi^j + \sum_{j=2}^{J}\Pi_c^j, \tag{6}$$

Similarly, the total external height can be derived as

$$H^t = \sum_{j=1}^{J} H^j, \tag{7}$$

This Miura-ori chain can be interpreted as J mechanical bits with the one-to-one correspondence between the stable configurations of each individual unit and the binary states. Therefore, a vector $\mathbf{A}$ ($\mathbf{A} \in \mathbb{R}^{J\times 1}$) with J binary elements can be derived to describe the configuration of the chain. For example, the configuration illustrated in the right panel in Figure S1(B) is interpreted as a vector shown on the right. In reverse, a digital vector can also be encoded to a physical configuration of the Miura-ori chain structure.

S2. Transition sequences with pre-defined actuation rules

For a single-unit cell, the configuration can be uniquely transformed by increasing or decreasing the height. Similarly, the multiple mechanical bits can also be rewritten with the actuation in the height direction. However, the origami chain structure may have multiple feasible configurations corresponding to one total external height. The actual transition depends on the current configuration as well as the loading path. Here, we introduce the optimization strategies to identify the transition sequences.

First of all, we define the actuation rule $\boldsymbol{\chi}$, $\boldsymbol{\chi} \in \mathbb{R}^{M\times 1}$, the mth element of this vector is denoted as χ_m, which takes either 1 ($\chi_m = 1$) or -1 ($\chi_m = -1$) values for increasing or decreasing the total external height of the structure, respectively. M represents the predefined actuation steps. Besides, the folding angles in m^{th} actuation step are denoted as $\boldsymbol{\theta}_m$. Based on the correspondence between the states of the mechanical bit and the stable configurations, the state vector in m^{th} actuation step, denoted as $\mathbf{A}_m$, can be derived with a sign function as

$$\mathbf{A}_m = \left(\text{sign}\left(\boldsymbol{\theta}_m\right)+1\right)/2. \tag{8}$$

Substituting the folding angles $\boldsymbol{\theta}_m$ into Equations (6) and (7), we have the total external height and the total potential energy of the current configuration, i.e., H_m^t and Π_m^t. Depending on the actuation rule χ_m, we slowly increase or decrease the external height by ΔH for $\chi_m = 1$ and $\chi_m = -1$, respectively:

$$H_m^t \rightarrow H_m^t + \chi_m \cdot \Delta H. \tag{9}$$

The folding angles corresponding to height H_m^t can be uniquely determined by searching the minimum potential energy in the neighborhood of the current folding angles. The corresponding states vector can be obtained with Equation (8). Once a new stable configuration is transformed, i.e., $\Pi^t = 0$, we update the states vector and start to execute the next actuation rule χ_{m+1}.

We keep the geometry parameters of every single unit cell to be the same (show in Equation (4)). However, in order to introduce the imperfection/asymmetry, we let the torsional

stiffness per unit length k^j be a random number around the design value (denoted as k), i.e., $k^j = \text{rand}[0.8, 1.2]k$. The equivalent stiffness per unit length is an order magnitude larger than the torsional stiffness per unit length in individual cells and is set as $k_c^j = 50k$. These parameters are used for all the simulations in the main text and the Supporting Information. For different SMO metamaterial, the crese stiffness are all randomly generated by this process.

As a simple case, the total number of the cells is set to be $J = 5$, and the initial configuration of all the unit cells is set at the '0' state. Define the actuation rule as $\boldsymbol{\chi} = [1 \quad 1 \quad 1 \quad 1]^T$, which represents a successive stretching of the origami chain structure with 4 transitions. The optimized potential energy landscape with respect to the external overall height is presented in Figure S1 (C). We find that the first and the last unit cell switch their configuration from state '0' to state '1' after the first actuation step. Then, the third one, the second one, and at last the fourth one gets transformed in succession with the actuation rules. Very rich transition sequences can be generated by executing this actuation process. From the mechanical memory point of view, the storage of the mechanical bits is rewritten by introducing the actuation rules, and a mechanical signal stream is produced. We demonstrate in the main text that this mechanical signal stream extends the dimensions of the inputs, and can be utilized as computing resources.

In Figure S1 (F), we present the potential energy landscape of the origami chain structure with the same initial configuration but with different actuation rules. In this simulation, after two configuration transitions by increasing the height, the structure is actuated with compression. Note that the paths do not overlap with each other when reversing the loading, instead, new configurations are generated. The corresponding state vectors are presented in Figure S1(G). It can be concluded that with different actuation rules, discriminative mechanical signal streams can be stimulated, which is however crucial for the separation of input states for reservoir computing.

Movie S1. Experimental Demonstration of Training the SMO Metamaterial to Recognize A Standard Digit 3.

Experimental demonstration of training the SMO metamaterial to recognize a standard digit 3 in Figure 2(C). Five encoding and transition processes are carried out for training the 5 rows of the image. All the procedures, including the encoding, experimental transitions, and the readout, are visually presented in the movie.

Movie S2. Experimental Demonstration of Training the SMO Metamaterial to Recognize A Standard Digit 3 with only one actuator.

Experimental demonstration of training the SMO metamaterial to recognize a standard digit 3 in Figure 2(C) with only one actuator. Resetting and transition processes are carried out for training the image. All the procedures, including resetting, experimental transitions, and the readout, are visually presented in the movie.
